# Smartphone-based activity tracking for spine patients: Current technology and future opportunities

**DOI:** 10.1016/j.wnsx.2023.100238

**Published:** 2023-10-01

**Authors:** Adam Leibold, Daniyal Mansoor Ali, James Harrop, Ashwini Sharan, Alexander R. Vaccaro, Ahilan Sivaganesan

**Affiliations:** a Department of Neurological Surgery, Thomas Jefferson University Hospital, Philadelphia, PA, USA; b Rothman Orthopaedic Institute, Jefferson Health, Philadelphia, PA, USA

**Keywords:** Smartphone-based activity tracking, Smartphone sensors, Mobile applications, Spine surgery, Patient-reported outcome measures (PROMs)

## Abstract

Activity trackers and wearables allow accurate determination of physical activity, basic vital parameters, and tracking of complex medical conditions. This review attempts to provide a roadmap for the development of these applications, outlining the basic tools available, how they can be combined, and what currently exists in the marketplace for spine patients.

Various types of sensors currently exist to measure distinct aspects of user movement. These include the accelerometer, gyroscope, magnetometer, barometer, global positioning system (GPS), Bluetooth and Wi-Fi, and microphone. Integration of data from these sensors allows detailed tracking of location and vectors of motion, resulting in accurate mobility assessments. These assessments can have great value for a variety of healthcare specialties, but perhaps none more so than spine surgery. Patient-reported outcomes (PROMs) are subject to bias and are difficult to track frequently - a problem that is ripe for disruption with the continued development of mobility technology.

Currently, multiple mobile applications exist as an extension of clinical care. These include Manage My Surgery (MMS), SOVINITY-e-Healthcare Services, eHealth System, Beiwe Smartphone Application, QS Access, 6WT, and the TUG app. These applications utilize sensor data to assess patient activity at baseline and postoperatively. The results are evaluated in conjunction with PROMs. However, these applications have not yet exploited the full potential of available sensors.

There is a need to develop smartphone applications that can accurately track the functional status and activity of spine patients, allowing a more quantitative assessment of outcomes, in contrast to legacy PROMs.

## Abbreviations

CMS: = Center for Medicare and Medicaid Services
DDD: = Degenerative disc disease
GPS: = Global positioning system
HCP: = Healthcare professionals
MMS: = Manage My Surgery
NDI: = Neck disability index
ODI: = Oswestry disability index
OFI: = Objective functional impairment
PD: = Parkinson's disease
PROMIS: = Patient-reported outcomes measurement information system
PROMs: = Patient-reported outcome measures
SF: = Short-form health survey
TUG: = Timed up and go
US: = United States
VAS: = Visual analogue scale
WD: = Walking distance
WT: = Walking test

## 1 Introduction

Technology has revolutionized how healthcare professionals (HCP) interact with patients. In response to the COVID-19 pandemic, the Center for Medicare and Medicaid Services (CMS) expanded payments and coverage of telemedicine services, and multiple large healthcare networks reported a 50-100-fold increase in telemedicine activity through 2020.^1^^,^^2^ With this wave of telemedicine comes an increasing need for tracking objective outcomes in the absence of in-person visits, making activity trackers and wearable all more important than ever.

Activity trackers and wearable allow for accurate determination of physical activity, basic vitals parameters, and even tracking of complex medical conditions such as heart failure and Parkinson's disease.^3^^,^^4^ There has been no specialty in medicine that is an exception to this phenomenon, including spine surgery. While it may be tempting to celebrate new technologies and their potential for improving the monitoring and care of patients, they are not always a net-positive for patients and HCPs. For example, telemedicine can be impersonal and carry uncertainties around certain aspects of the physical exam. Functional measurements through trackers and wearables can be useful, but their reliability, accuracy, and interpretation are not well defined. Additionally, HCPs may find it onerous to learn the details of these new systems and apply them to their practices, despite the potential advantages. Here we outline what smartphone-based tools are currently available for remote patient monitoring, what parameters those tools can measure, and how those tools can be integrated into patient care. We also detail how these tools are being used today in spine surgery, and what could be developed in the near future.

## 2 Smartphone sensors

We begin by providing a background for what types of sensors are readily available in mobile phones and what data this provides about a user's movements.

### 2.1 Accelerometer

An accelerometer is a circuit within the phone that is used to detect a change in velocity in any linear direction. In short, the accelerometer takes advantage of the piezoelectric effect in which a change in acceleration causes deformation or stress of microcrystals, and these crystals then emit a voltage which can be used to interpret direction and magnitude of the accelerative force. The accelerometers commonly found in smartphones can determine acceleration in both the X, Y, and Z axes.

### 2.2 Gyroscope

In contrast to the accelerometer in smartphones, gyroscopes are used to sense angular or rotational velocity and acceleration. The most obvious use of this is detecting our phones orientation, which allows our phones to switch from landscape to portrait mode automatically. In most cases, gyroscopic sensor data is used in concert with the accelerometer to create activity events and classify motion.

### 2.3 Magnetometer

Smartphones also contain a magnetometer used to sense the earth's magnetic field in the same way a compass does. Most commonly, this is used in the phone's compass and maps applications to sense the position and orientation of the phone with respect to North, South, East, and West. While accelerometers and gyroscopes are effective in sensing motion and acceleration in all directions, the magnetometer adds a global orientation. These sensors can provide an additional datapoint to determine the phone's orientation in space and are often integrated into applications sensing one's activity.

### 2.4 Barometer

Barometers sense changes in pressure. Traditional barometers become less accurate with decreasing size, which would historically have made it difficult to integrate them into smartphones. This was overcome by a group of Japanese scientists using a cantilever mechanism along with piezoelectronics to sense miniscule changes in pressure.^5^ These changes in pressure come mostly from weather or changes in elevation. In the sensing of movement, barometers are most useful for sensing changes in elevation. Climate and weather provide noise that can obscure this data. By using a network of barometer readings to reduce the noise created by changes in weather patterns, phones can use a barometer to accurately determine one's vertical position in space. This is important in sensing activity through changes in elevation (such as with stairs or uneven terrain).

### 2.5 Global positioning system (GPS)

The most familiar sensor used in phones is the GPS, which can sense a phone's location in relation to orbital satellites. GPS is a utility owned by the United States (US) Government and operated by the Air Force. Satellites use radio waves to communicate with one another and with a sensor in our phones, providing location and time information to our phones. Sensing one's change in location provides information such as distance traveled.

### 2.6 Bluetooth and Wi-Fi

Both Bluetooth and Wi-Fi sensors allow our phone to connect to a network, interacting with other connected devices in our area. By using co-location of Bluetooth sensors, you can infer interactions between people. Being connected to Wi-Fi and accessing the internet or interacting with other connected devices can also be used to infer social activity.

### 2.7 Microphone

Smartphones contain a microphone that can sense sound outside of simple telephone conversations. Some useful data that can be obtained is whether there is conversation happening, or whether a person is in a quiet or noisy environment. This has been used to track socialization, particularly in psychiatric disease monitoring.

## 3 Integration of data

The most basic level of activity sensing, and the first to be used in smartphones, was location tracking. GPS was able to classify certain locations such as “home” or “work.” The problem with using purely location-based data is that it can be inaccurate, nonspecific, and does not provide context. Someone at home taking a nap is different from that same person exercising in a home gym. As location-based data becomes more sophisticated, however, it can be used to determine more granular layers of location: is the subject next to a window, in the basement, or outside versus inside. Such information could be used to infer clues about mental health – those spending less time outside or near windows could potentially have a poorer mood, for example.

The next layer to be added to a person's location is locomotion. Accelerometers provide the foundation for locomotion sensing and have been used as the basis for detecting movement and activity in smartphones. These in combination with gyroscopes detecting angular movement, barometers for vertical movement, and GPS for overall change in location, can provide an accurate assessment of whether someone holding a smartphone is moving, and in what direction. Shoaib et al showed that each of the sensors mentioned above, except for the magnetometer, is capable of accurately capturing motion, and the performance improves when they are used in combination.^6^

### 3.1 Physiologic movements

Data integration can also allow us to infer the exact activity that a subject is partaking in (walking, running, sitting, climbing stairs) – not just his/her location and movement. Using the raw sensor data in combination with machine learning and neural networks, one can infer many types of physiologic movements, often with greater than 90% accuracy. Table 1 lists the approaches that have been used to convert raw sensor data into physiologic movements, as well as their effectiveness. Multiple groups have also released open-source databases that can be useful in the classification of movement activity for training models.^7^^,^^8^ By accurately estimating these physiologic movements, one can even begin to infer energy expenditure,^9^ posture, or ergonomics.^10^

**Table 1:** Approaches used to convert raw sensor data into physiologic movements and their effectiveness.

Reference	Sensor Data	Movements	Accuracy	Approach
**Voicu et al**^11^	Accelerometer, Gyroscope, magnetometer	Walking, Running, Sitting, Standing, Up Stairs, Downstairs	71–93%, Walking being most accurate, stairs least accurate	Multi-Layer Perceptron Neural Network
**Garcia-Gonzalez et al****^7^**	Accelerometer, gyroscope, magnetometer, GPS	Inactive, active, walking, driving	67.22% mean accuracy	Support Vector Machine Model
**Han et al**^12^	Accelerometer, gyroscope, GPS	Standing, walking, sitting, jogging, driving	81.2, 94.4% mean accuracy with Naïve Bayes, HMM, respectively	Naïve Bayes (NB), Hidden Markov Model (HMM)
**Pande et al****^9^**	Accelerometer, barometer	Energy Expenditure (EE), via walking, standing, stairs	96% Correlation with actual EE	Bagged Regression Decision Trees (DT) Model
**Pei et al**^13^	Accelerometer, gyroscope, magnetometer	Sitting, walking, fast walking, standing, turning slow, turning fast	92.9% accuracy	Least Square Support Vector Machine Model (LS-SVM)
**Gu et al**^14^	Barometer, Accelerometer	Still, walking, running, stairs, elevator	Average 90% accuracy for walking, running, still, 20–40% accuracy for stairs	Compared K Nearest Neighbor, Linear Discriminate Analysis, DT, NB, SVM, LS-SVM
**Mairittha et al**^15^	Accelerometer, Gyroscope	Classified 19 different activities (i.e., walking, running, cooking)	Increased activity recognition accuracy from 82% to 90%	Used convoluted and recurrent neural networks on device to train personalized ML models
**Guinness**^16^	GPS, Accelerometer	Walking, Running, Driving, Using Bus/Train	Able to detect with 97.5% accuracy using tuned Random Forest model	Compared DT, SVM, NB, BN, LR, ANN, and Kstar models

### 3.2 Use of smartphone activity data for disease monitoring

There are multiple disease states for which activity monitoring can add real value. From psychiatric diseases like schizophrenia and depression, to movement disorders like Parkinson's Disease (PD), clinicians often rely on subjective survey data. Neurologists use the PQD-39 to assess the severity of PD symptoms, primary care physicians use the PHQ-9 to screen for depression, and spine surgeons use metrics such as the visual analogue scale (VAS) and Oswestry Disability Index (ODI) to assess the severity of symptoms. These surveys are subject to inherent bias and to a patient's literacy.

Providing objective activity data can allow for more accurate monitoring. A clinical trial has shown that smartphones can provide activity data for PD patients that matches the accuracy of gold-standard clinical severity ratings.^17^ Machine learning techniques have also shown the ability to detect the severity of gait disturbances, thereby inferring the progression of PD.^4^ Patients with Alzheimer's disease can also be identified and monitored with similar activity measures.^18^ And in cancer patients, the ability to monitor activity and social interactions can provide valuable quality of life data.^19^^,^^20^ Lastly, activity and social interaction data can be used to monitor psychiatric diseases such as schizophrenia, depression, and bipolar disorder.^21^, ^22^, ^23^, ^24^ In spine surgery, with pain being a highly subjective measure and scales such as VAS or ODI having problems of bias, there is a clear need for more objective activity data in monitoring patient outcomes.

## 4 Mobile applications associated with spine surgery

Considering recent technological advancements, multiple mobile applications have been developed to assess outcomes in patients undergoing spine surgery. The detailed functionality of these applications is illustrated in Table 2.

**Table 2:** Detailed functionality of mobile applications used in spine surgery.

Title	Author	Journal	Mobile App	Metrics Measured
A Smartphone App With a Digital Care Pathway for Patients Undergoing Spine Surgery: Development and Feasibility Study	Madison Ponder et al.^25^	JMIR Perioper Med. 2020	ManageMySurgery (MMS)	The following questionnaires were filled at 6-weeks, 3-months, 6-months, and 12-months postoperatively: 1. Neck Disability Index 2. Percent pain reduction 3. Back Disability Index 4. Numerical pain assessment survey 5. Lumbar fusion approach assessment 6. Percent pain reduction 7. PROMIS-29
Postoperative monitoring with a mobile application after ambulatory lumbar discectomy: an effective tool for spine surgeons	Bertrand Debono et al.^26^	Eur Spine J. 2016	SOVINITY-e-Healthcare services (Cornebarrieu-France)	1. Visual analogue scale (VAS) for pain 2. Questionnaire regarding body temperature, painful voiding disorder, motor disorder, or a blood stain on the dressing
The Effectiveness and Safety of Utilizing Mobile Phone–Based Programs for Rehabilitation After Lumbar Spinal Surgery: Multicenter, Prospective Randomized Controlled Trial	Jingyi Hou et al.^27^	JMIR Mhealth Uhealth. 2019	eHealth system	The following outcomes were assessed at 3, 6, 12, and 24 months postoperatively: 1. Oswestry Disability Index (ODI) 2. Visual analog scale (VAS) for back pain 3. EuroQol-5 Dimension health questionnaire (EQ-5D) 4. 36-iten Short-Form Health Survey (SF-36)
Smartphone GPS signatures of patients undergoing spine surgery correlate with mobility and current gold standard outcome measures	Alessandro Boaro et al.^28^	J Neurosurg Spine. 2021	Beiwe smartphone application	The GPS sensor data was collected to report the following: 1. Overall distance covered (in km) 2. Radius of gyration (average radius travelled over a period of 1 day) 3. Diameter (largest distance between any two locations visited in a day) 4. Maximal distance from home (in km) 5. Time spent at home (in hours) 6. Number of locations visited (defined as a location where the phone has stationed for at least 5 min) 7. Proportion of time spent in significant locations (percentage of day) 8. Time of day spent moving (in hours) Patient-reported outcome measurements included: 1. Visual analog scale (VAS) for pain 2. Oswestry Disability Index (ODI) 3. Patient Reported Measures Information System 10 (PROMIS-10)
Using Smartphone-Based Accelerometer Data to Objectively Assess Outcomes in Spine Surgery	Gregory W Basil et al.^29^	Neurosurgery. 2021	QS Access (Quantified Self Labs, San Francisco, California)	The following data was obtained 24 months postoperatively: 1.Average daily distance walked (miles) 2.Flights climbed per day 3.Steps taken per day
Longitudinal smartphone-based self-assessment of objective functional impairment in patients undergoing surgery for lumbar degenerative disc disease: initial experience	Marketa Sosnova et al.^30^	Acta Neurochir (Wien) 2020	6WT app	Patient-reported outcome measurements included: 1. Numeric rating scale (NRS) leg pain 2. Core Outcome Measure Index (COMI) The 6WT app measured the following using global positioning system (GPS) coordinates: 1. Maximum walking distance (in meters) within 6 min [flat and level environment of at least 700 m distance was used]
Reliability of the 6-min walking test smartphone application	Martin *n*. Stienen et al.^31^	J Neurosurg Spine. 2019	6WT app	The following data was obtained by the 6WT app utilizing GPS coordinates: 1.Time to first symptoms (in seconds) 2.Distance to first symptoms (in meters) 3.6-minute walking distance (in meters) 4.Walking speed (slow <2 km/h, normal = 3.5 ± 1.5 km/h, fast >5 km/h)
Responsiveness of the self-measured 6-min walking test and the Timed Up and Go test in patients with degenerative lumbar disorders	Nicolai Maldaner et al.^32^	J Neurosurg Spine. 2021	6WT app TUG app	1. 6WT app = measured the 6-min walking distance (in meters) 2. TUG app = • Patients were seated on a chair with arms resting on the armrests • Patients had to get up and walk as fast as possible to a marked line at 3-m distance • Time (in seconds) was recorded • Patient had to turn, return to the chair and sit back down again after reaching the marked line •Main outcome = time between standing up and sitting down 3. The following patient-reported outcome measurements were obtained: • Zurich Claudication Questionnaire (ZCQ) • Core Outcome Measures Index for the back (COMI-back) • Visual analog scale (VAS) for low-back and lower-extremity pain
Objective Outcomes in Lateral Osteotomy Through Anterior-to-Psoas for Severe Adult Degenerative Spine Deformity Correction	Hasan S Ahmad et al.^33^	Cureus 2021	QS Access (Quantified Self Labs, San Francisco, CA)	1. Time series of steps taken per day 2. ODI score 3. PROMIS-pain interference score 4. PROMIS-physical function score
Distance to first symptoms measured by the 6-min walking test differentiates between treatment success and failure in patients with degenerative lumbar disorders	Anna M Zeitlberger et al.^34^	Eur Spine J. 2022	6WT app	1. 6-minute walking distance 2. Distance to first symptoms (DTFS) 3. Time to first symptoms (TTFS)
Assessment of the Minimum Clinically Important Difference in the Smartphone-based 6-min Walking Test After Surgery for Lumbar Degenerative Disc Disease	Anna M Zeitlberger et al.^35^	Spine 2021	6WT app	1. Raw 6-min walking distance 2. Age- and sex-adjusted z scores 3. Visual analog scale (VAS) for lower back and lower extremity pain 4. Zurich Claudication Questionnaire (ZCQ) for symptom severity and physical function 5. Core Outcome Measures Index (COMI) Back
Using smartphone-based accelerometers to gauge postoperative outcomes in patients with NPH: Implications for ambulatory monitoring	Annelise Claire Sprau et al.^36^	Surg Neurol Int. 2021	QS Access (Quantified Self Labs, San Francisco, CA)	1. Distance covered 2. Daily average steps walked

### 4.1 Manage My Surgery (MMS) app

The Manage My Surgery (MMS) application functions as an extension of clinical care. This app evaluates outcomes after surgery through the administration of surveys, rather than assessing or tracking activity measures. Questionnaires within the application capture baseline and post-operative patient-reported outcome measures (PROMs) at 6 weeks, 3 months, 6 months, and 12 months. Post-operative outcomes assessed include the 29-item patient-reported outcomes measurement information system (PROMIS-29), Oswestry disability index (ODI), neck disability index (NDI), numerical pain assessment, and percent pain reduction. By assessing outcomes at multiple time points, this application allows early identification and improved surgeon's response to potential red flags seen in patients. The MMS app has been successful in lowering overall anxiety, increased postoperative patient engagement and satisfaction, and better outcomes at a lower cost after spine surgery.^25^

### 4.2 SOVINITY-e-Healthcare Services app

The SOVINTY-e-Healthcare Services application monitors patients at baseline and post-operatively by the administration of multiple questionnaires. It assesses pain using the Visual Analogue Scale (VAS), body temperature, painful voiding disorder, motor disorder, or a blood stain on the dressing after surgery. Moreover, patient consultation with a surgeon between 30 and 45 days post-procedure, followed by a phone interview at the three-month mark, is used to assess morbidity after surgery. Different color codes are assigned based on patient responses to recovery indicators, resulting in reduced provider response time to potential red flags. Using this app proactively reduces hospitalization time, improves the effectiveness of outpatient care, and minimizes the burden on healthcare providers.^26^

### 4.3 eHealth system app

The eHealth System is a mobile-based rehabilitation program for patients that undergo lumbar spine surgery. This app assesses postoperative outcomes by administering questionnaires including ODI, VAS for back pain, the EuroQol 5-Dimension health questionnaire, and the 36-item Short-Form Health Survey (SF-36) to assess mental health. eHealth improves patient-provider communication and allows delivery of self-management interventions through its dual interface: a mobile phone-based interface for patients, and a web-based interface for doctors. Patients receive video instructions on how to conduct their rehabilitation remotely and they can communicate with their doctors through this system directly.^27^

### 4.4 Beiwe Smartphone Application

The Beiwe smartphone application is a research application, not available to the general public. This application can use smartphone sensor data in combination with survey data taken from research subjects. It has been used to combine GPS sensor data and PROMs obtained from patients undergoing spine surgery. The GPS measures overall distance covered (in kilometer), radius of gyration (average radius travelled in one day), diameter (largest distance between two locations in a day), maximum distance travelled from home (in kilometers), time spent at home, number of locations visited, proportion of time spent at each location, and time of day spent moving (in hours). PROM questionnaires administered include ODI, VAS for pain, and PROMIS-10. A comparison of smartphone GPS data to the current gold standard (PROMs) showed a strong correlation between the GPS-based features and VAS and PROMIS physical scores, while revealing a weak correlation to ODI and PROMIS mental scores. GPS-derived time-related variables (rather than distance) correlated better with patient-reported outcomes such as physical and performance status.^28^

### 4.5 QS access app

QS Access (Quantified Self Labs. San Francisco, California) is a free app that exports Apple Health (Apple Inc., Cupertino, CA) data. The smartphone sensor measures the average daily distance walked (miles), flights of stairs climbed per day, and steps taken per day. Apart from using sensor data, QS Access administers PROM questionnaires including ODI, PROMIS-pain interference, and PROMIS-physical function at multiple time points in the perioperative phase. In one study, patients showed decreased mobility up to 2 weeks postoperatively, as compared to sensor data in the 6 months prior to surgery.^33^ However, distance traveled, and steps taken improved significantly between 7 and 12 months after surgery. This application not only provided a more quantitative assessment of patient outcomes postoperatively, but also provided metrics that were not affected by recall bias, Hawthorne bias, and sampling bias (as is the case with PROMs).^33^

#### 4.5.1 6WT app

The 6WT smartphone application allows patients to identify objective functional impairment (OFI) in their home environment by using a 6-min walking distance test (6WD). This app uses GPS to measure the 6-min walking distance (in meters), time to first symptoms (in seconds), distance to first symptoms (in meters), and walking speed (slow <2 km/h, normal = 3.5 ± 1.5 km/h, fast >5 km/h). To test the reliability of the 6-min walking test (6WT), a total of 406 6WT measurements were obtained in one study.^31^ The results were reliable for measurements obtained in nature, in city environments without tall buildings, as well as straight, continuous, and stop-and-go walking patterns. However, in indoor areas, city environments with tall buildings, and rectangular walking courses, the measurements were unreliable.^31^

Utilization of this app for the longitudinal OFI assessment in patients with lumbar degenerative disc disease (DDD) before and during the first month after surgery provided physicians with additional information on the clinical course of each patient. The assessment of OFI provides a few advantages over questionnaire-based PROMs. First, by doing repeated physical tests in the home environment, patients feel empowered and their relationship with their physicians strengthens. Secondly, the longitudinal assessment of OFI results in faster detection of adverse events. Thirdly, change in 6WD assisted physicians in evaluating patient satisfaction/dissatisfaction with surgery. Lastly, once all the repeated measurements are transferred electronically to physicians, the decision-making process is significantly faster and optimized.^30^

### 4.6 TUG app

The TUG (Timed Up and Go) application is similar to the 6WT above, this is a simple application that can use smartphone data to time how long it takes one to stand and begin walking. In one study, the investigators performed this test during clinic visits. It only takes seconds and requires a chair and a 3-m walking space. Patients are asked to sit on a chair with their arms resting on the armrests. On command, patients get up and walk as fast as possible (without running) to a marked line at a 3-m distance while the examiner recorded the time (in seconds) using the TUG app. Once the patient reaches the marked line on the floor, he/she makes a turn, returns to the chair, and sits back down again. The time between standing up and sitting down is the main outcome. In comparison to the 6WT test, the TUG test showed inferior internal and external responsiveness.^32^

### 4.7 Back pain applications

In addition to a literature review of smartphone applications used to track patients with lumbar degenerative disease, we also performed a search for applications through commonly available online stores. The goal of this search was to determine what applications are available to the general public. We did so by typing “Back Pain” into the Apple App Store's search function and studying the top 25 applications. This less formal investigation was used to mirror what a back pain patient may do in the search for helpful tools and technology.

Out of the top 25 applications in the App Store, 21 (84%) primarily provide exercise and stretching content. Of the remaining four applications, 2/25 (8%) focus on symptom journaling. Lastly, one is an alarm reminding the user to stand, and another provides educational materials about back pain evidence-based management strategies.

Of note, none of the previously examined applications (from the literature review) were discoverable when searching for back pain solutions through the App Store. Only one of the App Store offerings provided activity tracking, using an estimation of caloric expenditure. Of these apps, 5 (20%) incorporated consultations for a fee with either a physician or physical therapist. The top 25 applications and their functions are listed in Table 3.

**Table 3:** Back pain-related mobile applications and their functions.

Application	Functions
Back pain exercises at home	Guided Exercises
Back Doctor | Pain Relief	Guided Exercises
PTX Therapy - 24/7 Pain Relief	Guided Exercises; Posture assessment; Symptom Journal
Hinge Health	Guided Exercises; PT consultation
Back Pain Yoga SSA	Guided Exercises
ROMWOD- mobility + recovery	Guided Exercises
Stretch & Flexibility at Home	Guided Exercises; Activity tracking
Scout for Back Pain	Guided Exercises; Symptom Journal; Treatment plan
Lower Back Pain Sciatica Spine	Guided Exercises
PainScale - Pain Tracker	Symptom Journal; physician report; Patient Education
10 Min Lower Back Therapy Workout	Guided Exercises
The Truth About Low Back Pain	Patient Education
MoovBuddy: Your Health Coach	Guided Exercises
Manage My Pain	Symptom Journal; Physician Report
Bend: Stretching and Flexibility	Guided Exercises
kiio	Guided Exercises; PT consultation
SimpleTherapy	Guided Exercises; Physician consultation
Curable	Guided Exercises; education; PT consultation; Symptom Journal
Posture by Muscle & Motion	Guided Exercises; eBook, Patient Education
Posture Reminder- Back Pain	Alarm
Atlas Low back Pain	Symptom Journal; Guided Exercises
Injurymap - Physiotherapy App	Guided Exercises; PT consultation; Symptom Journal
Back Pain Relief: Exercises	Guided Exercises
Bella's Lower Back Pain App	Guided Exercises; Symptom Journal
HEAL YOUR BACK	Guided Exercises

## 5 Gaps in current mobile applications and future directions

Although multiple sensors are present in new smartphones, existing mobile applications are yet to tap into their maximum potential for spine patients. Currently, readily used applications are taking advantage of either one, or a maximum of two sensors to track patient outcomes. A review of the literature reveals multiple methodologies for applying machine learning to multisensor data to provide rich insights about user movement, but this has yet to be implemented. Moreover, existing applications utilize different sets of PROMs to compare findings with sensor data. Different PROMs being used by various apps results in a lack of standardization and decreased comparability across platforms.

The state of art in mobile sensors and activity sensing is significantly ahead of what is being used in practice for spine patients. While advanced algorithms can detect detailed movement patterns, spine applications are limited to collecting simple data such as step counts or distance traveled. Additionally, applications marketed directly to individuals with back pain often do not utilize activity tracking at all. There is a need for applications, taking full advantage of existing sensors, to measure functional status for patients undergoing spine surgery as well as those pursuing conservative management.

## 6 Conclusion

Multiple types of smartphone sensors are readily available to provide accurate and nuanced information related to user movements. However, existing mobile applications have yet to take full advantage of these capabilities. New applications need to be developed that fill this gap, such that the functional status of spine patients can be quantified and tracked before and after surgical and non-surgical interventions. This review can serve as an initial roadmap for such efforts.

## Declaration of competing interest

The authors declare that they have no known competing financial interests or personal relationships that could have appeared to influence the work reported in this paper.
